# Supplementary material for: Decoding Graded Grip‐Force Intensity From fMRI Data Reveals a Transformation From Abstract to Effector‐ and Movement‐Specific Codes Prior to Execution
Source: Hum Brain Mapp. 2026 Oct 22;47(15):e70657. doi: 10.1002/hbm.70657 (PMC13687930; doi:10.1002/hbm.70657)
Supplement: Supplementary file 1 [file HBM-47-e70657-s001.docx]

**Decoding Graded Grip-Force Intensity from fMRI Data Reveals a Transformation from Abstract to Effector- and Movement-Specific Codes prior to Execution: Supplementary Materials**

**1. Control SVR Analyses & Xdec: Color-based Levels Decoding**

To test whether force intensities were represented in an abstract, effector-independent format during the first delay period—and to ensure that the results of the main decoding analysis were not biased by the specifics of the employed FIR model or by the effector-specific partitioning of beta estimates in SVR models—we provided converging evidence using an alternative FIR and conducted supplementary SVRs and cross-decoding analyses based on the color of the visually cued force level (i.e., cyan or red), rather than the hand prepared during the second delay. Each of these analyses employed a single FIR model, similar to that used in the main decoding. However, in this case, the first-level GLM represented each combination of grip-force intensity and force-level color (rather than intensity and effector), capturing each intensity as displayed in the *grip-force cue*. This resulted in a first-level GLM with 688 regressors (8 conditions × 20 time bins × 4 runs), complemented by six realignment parameters and five principal components. Beta estimates for the four force intensities were entered into two corresponding SVR models (one per color), and the same four-fold leave-one-run-out cross-validation scheme used in the main decoding analysis was applied, generating 20 prediction-accuracy maps for analysis. For the simple SVR analyses, decoding of force intensities was performed independently for each color (i.e., two separate analyses). For cross-decoding, a single SVR model was trained on beta estimates reflecting presentation of the force levels in cyan and tested on those in red, and vice versa. After normalization and smoothing, prediction accuracy maps were entered into two second-level analyses to test for above-chance decoding using SPM's flexible factorial design implementation of an ANOVA. The first second-level design included maps from both levels’ color and comprised two factors—one for color (cyan and red)—while the second design included a single between-color factor. Each factor included 20 levels corresponding to the time bins (t1–t20). T-contrasts were used to assess above-chance decoding in the second half of the first delay period, as in the main analysis.

Convincingly, when testing for color independent encoding of force intensities (first design), a t-contrast across colors is conducted and reveals activation patterns similar to those found in the main analysis during Delay 1 (see **Figure 3A**), with above-chance decoding in the PCu/SPL, at *p* < 0.05 FWE-corrected (see **Figure S1A** and **Table S1**). Comparable results were also found in the cross-decoding analysis, where above-chance decoding corroborated a color-independent encoding of force intensity in PCu/SPL (see **Figure S1B**, and **Table S1**). Together, these analyses indicates that the parametric code found in the PCu/SPL reflects the encoding of graded force intensities regardless the force levels’ color. In contrast, the main decoding show that force intensities are encoded regardless the effector.

Together, these results indicate that above-chance decoding during the first delay does not relay on the levels’ color or effector coding, yet parametric codes in the PCu/SPL reflect encoding of force intensities for later execution. Findings of the SVRs, cross-decoding analyses and the main decoding support the notion that graded force-intensities are represented in the PCu/SPL in an abstract format during the first delay period.

**Figure S1** Results of time resolved SVR and cross-decoding analyses **A.** Displays brain regions that parametrically code grip-force intensities during the first delay period (Delay 1). The grey panel indicates encoding of force intensities regardless color of the force level (Delay 1). Brain regions with above-chance prediction accuracy are revealed by t-contrasts testing the respective periods against zero, at *p* < 0.05, FWE-corrected. In Delay 1, two clusters were found in the PCu/SPL and r-pIPS (first column). **B.** Displays results of the Xdec for the first delay period. Brain regions with above-chance prediction accuracy are revealed by t-contrasts against zero, at *p* < 0.05, FWE-corrected.

| Cluster size | Anatomical Region | Peak MNI coordinates | | |  |
| --- | --- | --- | --- | --- | --- |
|  |  | x | y | z | z-score |
| **Level colour based SVR** | | | | | |
| 374 | Right pIPS | 40 | -44 | 52 | 6.08 |
|  | PCu/SPL | 7 | -62 | 52 | 5.07 |
|  |  | -12 | -60 | 44 | 4.70 |
| **Level colour based Xdec** | | | | | |
| 140 | PCu/SPL | 12 | -60 | 52 | 6.16 |
|  |  | -10 | -62 | 54 | 4.88 |
| 19 | Right pIPS | 52 | -22 | 48 | 4.79 |

**Table S1** T-contrast of level color-based SVR and Xdec, testing regions with above-chance decoding in the first delay period, at *p* < 0.05, FWE corrected.

**2. Control: Parametric Contrast on Permutation testing**

**Figure S2** **A.** Results of parametric contrast over prediction accuracy maps from the label permutation tests (see Methods), during preparation with the left hand (upper display, green background) the right hand (lower display, green background), and before preparation (grey background). Parametric contrasts to test for parametric modulation in prediction accuracy due to the increase in level of order of the permuted labels (i.e., permutation difference) are presented across the same time periods as in the main analysis: first delay period (Delay 1), second delay period (Delay 2) and motor execution period (Execution). Above-chance prediction accuracy clusters are displayed at p < 0.05, FWE-corrected (see Table S1). As in the main decoding analysis, during Delay 1, a cluster in PCu/SPL was revealed. The results of the main analysis were corroborated also for the second Delay 2, by showing contralateral pIPS, aIPS, S1, PMd, and SMA. Finally, t-contrasts on the Execution revealed contralateral M1s and S1s (third column). **B.** Results of permutation testing in which the same SVR analysis was performed with permuted labels of the data. Prediction accuracy values were extracted from the peak voxels of the six most representative clusters found in the main analysis: r-pIPS [x=26, y=--62, z=50], l-pIPS [x=-28, y=--66, z=52], r-aIPS [x=-36, y=-42, z=58], l-aIPS [x=-38, y=-42, z=58], r-PMd [x=30, y=-12, z=56] and l-PMd [x=-38, y=--8, z=62]. The time-course represents three prediction accuracy values obtained by averaging prediction accuracy values of twenty time-bins in correspondence of the three time-periods (tested in the main analysis). The divergence of the permutations from the linear order of grip-force levels is expressed as distance in rank order. As expected, the divergence from the original order reduces the performance of the SVR.

| Cluster size | Anatomical Region | Peak MNI coordinates | | |  |
| --- | --- | --- | --- | --- | --- |
|  |  | x | y | z | z-score |
| **First delay Period** | | | | | |
| 245 | PCu/SPL | -8 | -68 | 58 | 5.09 |
|  |  | -14 | -78 | 42 | 5.06 |
| 17 | Left aIPS | -52 | -34 | 50 | 4.73 |
| 11 | Right iPS | 26 | -42 | 72 | 4.56 |
| **Second delay Period, left hand** | | | | | |
| 6302 | Right pIPS | 20 | -60 | 60 | 6.81 |
|  | Right aIPS | 42 | -40 | 56 | 6.64 |
|  | SMA | 0 | 2 | 68 | 6.60 |
|  | Right PMd | 38 | -10 | 68 | 6.55 |
| 523 | Left IPL | -62 | -26 | 34 | 6.33 |
| 1061 | PCu/SPL | -18 | -58 | 58 | 6.11 |
| 157 | Right dlPFC | 34 | 48 | 30 | 5.11 |
| **Second delay Period, right hand** | | | | | |
| 587 | Left S1 | -60 | -22 | 32 | 6.02 |
| 867 | Left PMd | -36 | -4 | 62 | 5.79 |
| 355 | SMA | 4 | -2 | 66 | 5.62 |
| 12 | Left pIPS | -30 | -66 | 54 | 4.67 |
| 6 | Left aIPS | -40 | -48 | 60 | 4.57 |
| 3 | Left dlPFC | -34 | 46 | 20 | 4.55 |
| **Motor execution Period, left hand** | | | | | |
| 34317 | Right M1 | 32 | -26 | 62 | 18.66 |
| **Motor execution Period, right hand** | | | | | |
| 74 | Left M1 | -30 | -26 | 56 | 19.36 |

**Table S2** T-contrast of the parametric permutation testing revealed regions that exhibited above-chance prediction accuracy across the cue period, first and second delay periods, and motor execution period, at p < 0.05, FWE corrected. The l-EBA was found at a p < 0. 001 uncorrected level.

**3. Control: SVR based on Mean Reproduced Grip-force**

**Figure S3** Results of time-resolved support vector regression analysis base on mean reproduced grip-force values as decoding labels **A.** Brain regions showing parametric coding of grip-force intensity during the first delay period (Delay 1), second delay period (Delay 2), and motor execution period (Execution), using the mean reproduced force values for each force level, hand, and participant as SVR labels. The decoding scheme and statistical procedures were identical to those of the main analysis, with the only difference being the use of behavioural reproduced forces instead of Fechner-corrected force intensities as labels. The grey panel represents encoding of force intensity independent of the effector used during Delay 1; the upper green panel represents preparation and execution with the left hand the lower blue panel represents preparation and execution with the right hand. Brain regions showing above-chance decoding were identified using t-contrasts testing respective periods against zero, at *p* < 0.05, FWE-corrected. During Delay 1 (first column), clusters were identified in the PCu/SPL. During Delay 2 (second column), lateralized clusters were observed in the pIPS, aIPS, S1, PMd, and the SMA in the hemisphere contralateral to the prepared hand, together with the PCu/SPL. Finally, during Execution (third column), t-contrasts revealed contralateral and ipsilateral clusters in M1. The spatial distribution of decoding clusters closely resembled the results obtained using Fechner-corrected force intensity labels, supporting the robustness of the findings with respect to the definition of SVR labels.

| Cluster size | Anatomical Region | Peak MNI coordinates | | |  |
| --- | --- | --- | --- | --- | --- |
|  |  | x | y | z | z-score |
| **First delay Period** | | | | | |
| 57 | PCu/SPL | -14 | -62 | 54 | 4.83 |
| **Second delay Period, left hand** | | | | | |
| 6799 | SMA | 2 | 0 | 60 | 7.31 |
|  | Right aIPS | 42 | -40 | 60 | 6.68 |
|  | Right pIPS | 36 | -46 | 64 | 6.50 |
|  | PCu/SPL | -12 | -62 | 62 | 6.23 |
|  | Righ PMd | 26 | -10 | 60 | 5.65 |
| 2581 | Left IPL | -60 | -26 | 34 | 7.10 |
|  | Left pIPS | -22 | -54 | 62 | 6.90 |
| 1094 | Left dlPFC | -38 | 54 | 24 | 5.57 |
| 629 | Right dlPFC | 28 | 50 | 34 | 5.54 |
| 143 | Left PMd | -48 | 6 | 48 | 5.13 |
| **Second delay Period, right hand** | | | | | |
| 4496 | Left PMd | -36 | -10 | 64 | 7.19 |
|  | Left aIPS | -40 | -40 | 56 | 6.23 |
|  | Left S1 | -60 | -40 | 56 | 6.14 |
|  | Left pIPS | -28 | -60 | 56 | 5.71 |
|  | PCu/SPL | -10 | -62 | 56 | 4.93 |
| 2050 | SMA | 2 | -4 | 64 | 6.30 |
|  | Right PMd | 36 | -16 | 56 |  |
| 506 | Left PMv | -56 | 4 | 38 | 5.15 |
| 128 | Left dlPFC | -32 | 46 | 40 | 5.29 |
| **Motor execution Period, left hand** | | | | | |
| 560650 | Right M1 | 32 | -26 | 62 | 18.10 |
| **Motor execution Period, right hand** | | | | | |
| 267620 | Left M1 | -32 | -24 | 56 | 17.53 |

**Table S3** T-contrasts identifying regions showing above-chance prediction accuracy in the control analysis using mean reproduced grip-force values as SVR labels, at *p* < 0.05, FWE corrected.

**4. Control: SVR based on Linearly Scaled (Non-Fechner-Corrected) Grip-force Intensity Labels**

**Figure S4** Results of time-resolved support vector regression analysis based on linearly scaled (non-Fechner-corrected) grip-force intensities as decoding labels **A.** Brain regions showing parametric coding of grip-force intensity during the first delay period (Delay 1), second delay period (Delay 2), and motor execution period (Execution), using linearly scaled SVR labels corresponding to the instructed grip-force intensities (−1.5, −0.5, 0.5, and 1.5) without Fechner correction. The decoding scheme and statistical procedures were identical to those of the main analysis, with the only difference being the use of linearly scaled labels instead of Fechner-corrected labels. The grey panel represents encoding of force intensity independent of the effector used during Delay 1; the upper green panel represents preparation with the left hand during Delay 2; the lower blue panel represents preparation with the right hand during Delay 2. Brain regions showing above-chance prediction accuracy were identified using t-contrasts testing decoding accuracy against zero, at p < 0.05, FWE-corrected. During Delay 1 (first column), clusters were identified in the PCu/SPL. During Delay 2 (second column), lateralized clusters were observed in the pIPS, aIPS, S1, PMd, and SMA in the hemisphere contralateral to the prepared hand, together with the PCu/SPL. Finally, during Execution (third column), t-contrasts revealed contralateral and ipsilateral clusters in M1. The spatial distribution of decoding clusters closely resembled the results obtained using Fechner-corrected force intensity labels, demonstrating that the reported findings are robust to the specific scaling applied to the instructed force intensities.

| Cluster size | Anatomical Region | Peak MNI coordinates | | |  |
| --- | --- | --- | --- | --- | --- |
|  |  | x | y | z | z-score |
| **First delay Period** | | | | | |
| 157 | PCu/SPL | 10 | -66 | 50 | 5.14 |
| 109 | PCu/SPL | -14 | -62 | 56 | 4.95 |
| **Second delay Period, left hand** | | | | | |
| 9094 | Right pIPS | 26 | -62 | 50 | 7.60 |
|  | Right aIPS | 36 | -48 | 64 | 7.24 |
|  | SMA | 0 | 0 | 64 | 6.77 |
|  | Right PMd | 28 | -8 | 64 | 5.86 |
| 2760 | Left IPL | -58 | -26 | 32 | 7.40 |
|  | PCu/SPL | -14 | -60 | 58 | 5.86 |
| 971 | Left dlPFC | -22 | 50 | 36 | 5.86 |
| 439 | Right PMd | -50 | 4 | 42 | 5.70 |
| 157 | Right dlPFC | 34 | 48 | 30 | 5.11 |
| **Second delay Period, right hand** | | | | | |
| 5599 | Left pIPS | -30 | -64 | 52 | 6.95 |
|  | Left PMd | -36 | -8 | 64 | 6.77 |
|  | Left S1 | -62 | -18 | 30 | 6.37 |
|  | Left aIPS | -42 | -38 | 60 | 5.85 |
|  | PCu/SPL | -10 | -66 | 54 | 5.11 |
| 1299 | SMA | 2 | -4 | 66 | 6.63 |
|  | Right PMd | 26 | -6 | 74 | 5.72 |
| 190 | Left dlPFC | 40 | 34 | 30 | 5.40 |
| **Motor execution Period, left hand** | | | | | |
| 386710 | Right M1 | 32 | -26 | 62 | 19.64 |
| **Motor execution Period, right hand** | | | | | |
| 259890 | Left M1 | -34 | -24 | 58 | 17.76 |

**Table S4** T-contrasts from the time-resolved SVR analysis using linearly scaled SVR labels, at *p* < 0.05, FWE corrected.

**5. HRF convolved GLM: Univariate Parametric Modulation during Motor Execution**

**Figure S5 A.** Results of t-contrasts on contrast images reflecting univariate parametric modulation in brain activation levels during motor execution (E) with the left (green) and right hand (blue). Contrast images were obtained by parametrically weighting beta estimates derived from an HRF convolved GLM model. Significant parametric modulation was observed in the contralateral right M1 and left M1 (at p < 0.05, FWE-corrected) (see Table S4).

| **Motor execution Period, left hand** | | | | | |
| --- | --- | --- | --- | --- | --- |
| 607 | Right M1 | 36 | -24 | 58 | 10.21 |
| **Motor execution Period, right hand** | | | | | |
| 46 | Right M1 | -40 | -20 | 18 | 7.86 |

**Table S5** Regions that exhibit univariate parametric activity modulation during motor execution, at p < 0.05, FWE corrected.
